# LncRNA OIP5-AS1 suppresses lung adenocarcinoma progression and modulates macrophage polarization through the miR-429/DOCK4 regulatory axis

**DOI:** 10.3389/fphar.2025.1569644

**Published:** 2025-05-20

**Authors:** Yihan Liu, Yuhua Wang, Long Cui, Runze Li, Dan Xiao

**Affiliations:** ^1^ Graduate School, Heilongjiang University of Chinese Medicine, Harbin, China; ^2^ School of Marxism, Qiqihar Medical University, Qiqihar, China; ^3^ Department of Oncology, Qiqihar Hospital of Chinese Medicine, Qiqihar, China; ^4^ National and Local Joint Engineering Laboratory for Synthesis Transformation and Separation of Extreme Environmental Nutrients, Harbin Institute of Technology, Harbin, China; ^5^ Zhengzhou Research Institute, Harbin Institute of Technology, Zhengzhou, China

**Keywords:** macrophage polarization, OIP5-AS1, miR-429, DOCK4, lung adenocarcinoma

## Abstract

**Background:**

Macrophage polarization plays a pivotal role in shaping the tumor microenvironment and influencing cancer progression. Long non-coding RNAs (lncRNAs) have emerged as important regulators of this process. This study investigated the role of lncRNA OIP5-AS1 in lung adenocarcinoma (LUAD) progression and its involvement in macrophage polarization.

**Methods:**

The expression of OIP5-AS1 in LUAD tissues and its association with patient prognosis were analyzed. Functional assays, including cell proliferation, migration, invasion, and cell cycle analysis, were conducted in LUAD cell lines. Bioinformatics prediction, luciferase reporter assays, and RNA immunoprecipitation (RIP) were used to explore the interaction among OIP5-AS1, miR-429, and DOCK4. Macrophage polarization and migratory capacity were assessed following manipulation of OIP5-AS1, miR-429, and DOCK4 expression.

**Results:**

OIP5-AS1 expression was significantly decreased in LUAD tissues and associated with poor survival. Overexpression of OIP5-AS1 inhibited LUAD cell proliferation, migration, and invasion, induced G1 phase arrest, and suppressed tumor growth *in vitro*. Mechanistically, OIP5-AS1 functioned as a molecular sponge for miR-429, regulating DOCK4 expression. Overexpression of miR-429 or knockdown of DOCK4 reversed the effects of OIP5-AS1 on macrophage polarization markers and restored macrophage migration.

**Conclusion:**

OIP5-AS1 modulates macrophage polarization through the miR-429/DOCK4 axis and inhibits LUAD cell progression. This regulatory pathway may influence the tumor immune microenvironment and represent a potential therapeutic target in LUAD.

## Introduction

Lung cancer remains a leading cause of cancer-related deaths globally, with lung adenocarcinoma (LUAD) as the most common subtype (Lee and Kazerooni, 2022; Wei et al., 2023; Li et al., 2024). Recent studies have provided updated data on lung cancer incidence and mortality trends, reaffirming its significant global burden (Siegel et al., 2024; Liu X. et al., 2024; Xu and Lu, 2024). Although recent advancements in immunotherapy and targeted therapies, along with the identification of structural features and molecular targets in lung cancer, have improved treatment outcomes for LUAD, the overall prognosis remains poor, with high rates of recurrence and metastasis (Lovly, 2022; Song et al., 2023; Wu et al., 2023). Therefore, a better understanding of the molecular mechanisms driving LUAD progression and the identification of novel therapeutic targets are essential.

Long non-coding RNAs (lncRNAs) are endogenous RNA molecules longer than 200 nucleotides that do not encode proteins but regulate gene expression, often acting as “molecular sponges” for microRNAs (miRNAs) (McCabe and Rasmussen, 2021). LncRNAs play crucial roles in tumorigenesis, cancer progression, and resistance to therapy (Peng et al., 2017; Luo et al., 2021). Among these, Opa-interacting protein five antisense RNA 1 (OIP5-AS1) has emerged as a multifunctional regulator with context-dependent roles in different cancer types. In endometrial cancer, distinct regulatory mechanisms have been reported: one study demonstrated that OIP5-AS1 sponges miR-200c-3p to modulate the PTEN/AKT pathway, thereby inhibiting cell proliferation and invasion (Liu et al., 2021), while another showed that OIP5-AS1 targets miR-152-3p to upregulate SLC7A5, promoting tumor cell development (Liang M. et al., 2021). These findings underscore the complex and context-specific functions of OIP5-AS1, which may vary depending on the interacting miRNAs and downstream targets.

miR-429, a member of the miR-200 family, exhibits dual regulatory roles in cancer, acting as both a promoter and suppressor of tumorigenesis (Liu et al., 2018). It has been implicated in several critical signaling pathways across various cancers, including the NF-κB, Ras/MEK/ERK, and PI3K/AKT pathways (Fan et al., 2017; Guo et al., 2018; Zhao Z. et al., 2021). While the functions of miR-429 have been explored in multiple cancer types, its specific role in LUAD and its interactions with lncRNAs remain largely unexplored.

DOCK4, part of the Rho GTPase exchange factor family, has been linked to cancer progression (Zhao Q. et al., 2021). Single-cell and bulk RNA sequencing analyses have identified 12 genes, including DOCK4, that are associated with M2 macrophage polarization and angiogenesis, suggesting their potential as prognostic markers in LUAD (Liu A. et al., 2024). However, the role of DOCK4 in regulating macrophage polarization and its underlying mechanisms remains largely unclear.

Tumor-associated macrophages (TAMs) are the most abundant immune cells in the tumor microenvironment (TME) and are strongly linked to tumor proliferation, metastasis, invasion, and angiogenesis (Wang J. et al., 2022; Sedighzadeh et al., 2021; Sun et al., 2021). Depending on signals from the TME, TAMs can polarize into M1 macrophages, which exhibit anti-tumor activity, or M2 macrophages, which promote tumor progression (Boutilier and Elsawa, 2021). Increasing evidence suggests that non-coding RNAs play a pivotal role in reshaping the TME and driving tumor metastasis (Mohapatra et al., 2021). Whether OIP5-AS1 contributes to LUAD progression by modulating macrophage polarization, however, has yet to be fully elucidated.

This study investigated the expression of OIP5-AS1 in LUAD and its effects on the biological behavior of LUAD cell lines H358 and H1650 *in vitro*. It specifically examined whether OIP5-AS1 regulates macrophage polarization and tumor progression by acting as a competing endogenous RNA (ceRNA) that interacts with miR-429 to modulate DOCK4 expression. For the first time, the role of the OIP5-AS1/miR-429/DOCK4 regulatory axis in LUAD was revealed, with a particular emphasis on its impact on macrophage polarization. These findings provide new insights into the molecular mechanisms driving LUAD progression.

## Materials and methods

### TCGA and GEO dataset analysis

Gene expression data were obtained from the TCGA database and the GSE140343 and GSE229705 datasets. Differential expression analysis was performed using the limma package in R. TPM normalized data were log2 transformed (log2 (TPM+1)) before analysis. Adjusted p-values (FDR) were calculated to correct for multiple testing. The significance indicated in the boxplot visualization was based on the limma adjusted p-values (FDR). We utilized ggplot2 and ggpubr exclusively for plotting and statistical visualization. Samples were categorized into high- and low-expression groups based on the median expression level of the gene. Survival analysis was conducted using the R package survival, and Kaplan-Meier survival curves were generated with survminer.

Survival analysis was performed using the R survival package (survdiff () function), applying a standard log-rank test to compare the overall survival distributions between the two groups. The resulting p-value reflects the difference across the entire follow-up period. In the Kaplan–Meier plot, horizontal and vertical lines (surv.median.line = “hv”) indicate each group’s median survival time, serving purely as a visual reference. Differences in clinical characteristics between groups were analyzed and visualized using the R package ggpubr.

### Cell line, cell culture and transfection

All cell lines used in this study (H358, H1650, PC-9, H1755, and BEAS-2B) were obtained from the Institute of Cell Research, Chinese Academy of Sciences (Shanghai, China). H358, H1650, PC-9, and H1755 cells were maintained in RPMI 1640 medium (Gibco, United States) at 37°C in a humidified 5% CO2 incubator.BEAS-2B cells were maintained in LHC-9 medium (Gibco, United States) without serum, following the recommended protocol, to prevent spontaneous differentiation. The culture medium was refreshed every 48 h, and cells were not passaged beyond passage 10 to ensure phenotypic stability.

Short hairpin RNAs (shRNAs) targeting OIP5-AS1 and DOCK4, as well as the negative control (sh-NC), were commercially synthesized and cloned into pLKO.1 lentiviral vectors (GeneChem, Shanghai, China). The specific target sequences for DOCK4 shRNA, OIP5-AS1 shRNA are provided in Supplementary Table S1. The pcDNA3.1-OIP5-AS1 overexpression plasmid and its control vector (pcDNA3.1-empty) were obtained from GeneChem (Shanghai, China). Transfections were carried out using Lipofectamine 3,000 (Invitrogen, CA, United States) in accordance with the instructions provided by the manufacturer.

### Real-time PCR

Total RNA was extracted from cells using TRIzol reagent (Invitrogen, CA, US). The extracted RNA was reverse-transcribed into complementary DNA (cDNA) using the High-Capacity cDNA Reverse Transcription Kit (Applied Biosystems, Carlsbad, CA, United States). Real-time PCR was performed on an ABI7500 Real-Time PCR System (Applied Biosystems, Carlsbad, CA, United States) with SYBR Green I dye to quantify RNA expression levels. Relative expression levels were calculated by 2^−ΔΔCq^ method. Primer sequences used are provided in Supplementary Table S2.

### CCK8

Cell viability was assessed using the CCK-8 assay kit (Beyotime, Shanghai, China). Similar methodologies have been previously employed (Qiu et al., 2024). Cells were seeded at a density of 1,000 cells per well in 96-well plates and incubated under standard culture conditions. At 24, 48 and 72 h, CCK-8 reagent was added to each well, and cell viability was measured using a microplate reader at a wavelength of 450 nm.

### Wound healing assay

LUAD cells were seeded in 6-well plates and allowed to grow to 90%–100% confluence. A scratch was created in the cell monolayer using a 200 μL pipette tip. After washing the wells with PBS (Sigma Aldrich, St. Louis, United States), images were captured at 0 and 48 h under a microscope.

### Transwell assay

Cells were seeded into the upper chamber of Transwell inserts pre-coated with Matrigel (Corning, United States), while the lower chamber was filled with complete medium. After 48 h of incubation, non-migrated cells were removed. The migrated cells were stained with 0.1% crystal violet and counted under a light microscope.

### Flow cytometry assay

Cells were collected and washed with pre-cooled PBS, then fixed at 4°C overnight. After fixation, the cells were incubated with PI staining solution in the dark for 15 min. Cell cycle distribution, including the G1, S, and G2 phases, was analyzed by flow cytometry.

### THP-1 cell differentiation and co-culture

THP-1 cells were seeded in a 6-well plate at a density of 1 × 10^6^ cells/well and cultured with 200 nM PMA (Sigma-Aldrich, St. Louis, United States). After 48 h of stimulation, the PMA-containing medium was replaced with fresh culture medium. THP-1 cells were then co-cultured with conditioned medium for 48 h.

### Conditioned medium (CM) preparation

LUAD cells were seeded in 6-well plates at 5 × 10^5^ cells/well and transfected with OIP5-AS1/miR-429/DOCK4 overexpression or knockdown constructs. After 24 h, cells were washed and incubated in serum-free medium for 24 h to generate CM. Supernatants were collected, centrifuged (1,000 × g, 10 min, 4°C) to remove cellular debris, and filtered through a 0.22-μm membrane.

#### Macrophage polarization assay

Differentiated THP-1 macrophages were treated with CM for 48 h. Total RNA was then extracted from cells and analyzed by RT-PCR to evaluate the expression of macrophage polarization markers.

### Cell migration assay

THP-1 macrophages (1 × 10^5^ cells/well) in serum-free medium were seeded in the upper chamber (8 μm pore, Corning). The lower chamber contained CM from LUAD cells or control medium (serum-free RPMI-1640). After 24 h at 37°C, non-migrated cells on the upper membrane surface were removed with a cotton swab. Migrated macrophages on the lower membrane surface were fixed with 4% paraformaldehyde, stained with 0.1% crystal violet, and imaged under a light microscope (×200magnification). Cells in five random fields per insert were counted using ImageJ.

### Luciferase reporter assay

The binding sites between miR-429 and OIP5-AS1 or DOCK4 were predicted using the StarBase database (http://starbase.sysu.edu.cn/index.php). Wild-type reporter constructs (OIP5-AS1-WT and DOCK4-WT) and mutant versions (OIP5-AS1-MUT and DOCK4-MUT) were commercially synthesized by Sangon Biotech (Shanghai, China) using the pGL3-control vector. Mutations were introduced via site-directed mutagenesis to disrupt predicted miR-429 binding sites.

For the luciferase assay, H358 and H1650 cells were co-transfected with the reporter constructs together with miR-429 mimic, miR-429 inhibitor, or respective negative controls (NCs). Luciferase activities were determined 24 h post-transfection using the dual-luciferase reporter assay system.

### RNA immunoprecipitation (RIP)

The interaction among OIP5-AS1, miR-429, and DOCK4 was examined using RIP kit (Bersinbio, Guangzhou, China). Cells were collected and resuspended in RIP lysis buffer containing protease inhibitors. The cell lysates were incubated overnight with antibodies against Ago2 or IgG. RNA-protein complexes were then captured using protein A/G magnetic beads. After digestion with proteinase K, RNA was extracted from the complexes for subsequent analysis.

### Statistical analysis

All statistical analyses were performed using SPSS 23.0 (IBM Corp., Armonk, NY, United States). Differences between two groups were assessed with the t-test, while comparisons among multiple groups were conducted using one-way analysis of variance (ANOVA). P < 0.05 was considered statistically significant.

## Results

### Expression and clinical characterization of OIP5-AS1 in LUAD

**FIGURE 1:**
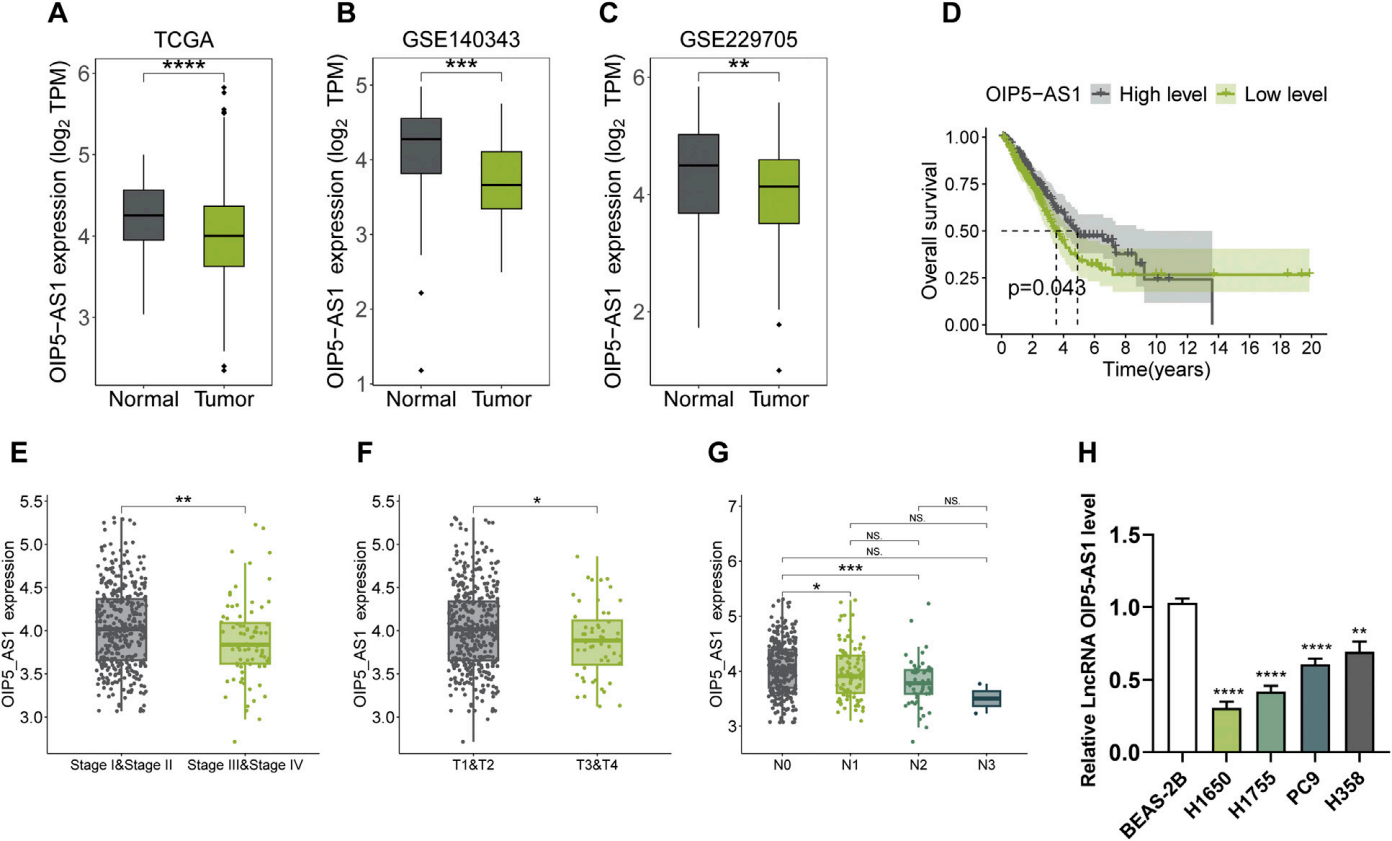
Expression of OIP5-AS1 in LUAD and its clinical significance. **(A–C)** OIP5-AS1 transcription levels in LUAD tissues compared to normal tissues based on analyses of the TCGA dataset **(A)**, GSE140343 dataset **(B)**, and GSE229705 dataset **(C)**; **(D)** Kaplan-Meier survival analysis of OIP5-AS1 expression in LUAD patients from the TCGA dataset (cut-off: median, 50th percentile); **(E)** OIP5-AS1 expression across different TNM stages in LUAD patients; **(F)** OIP5-AS1 expression levels stratified by tumor size in LUAD patients; **(G)** OIP5-AS1 expression level in patients with or without lymph node metastasis; **(H)** OIP5-AS1 expression in normal human lung epithelial cells (BEAS-2B) compared to lung cancer cell lines. (*p < 0.05, **p < 0.01, **p < 0.01, ****p < 0.0001, NS: not significant.)

Bioinformatics analysis revealed a significant downregulation of OIP5-AS1 in LUAD tissues compared to normal tissues, as shown in both TCGA and GEO datasets (GSE140343 and GSE229705) (Figures 1A–C). Kaplan-Meier survival analysis indicated that lower OIP5-AS1 expression was significantly associated with poorer overall survival (Figure 1D). In addition, low OIP5-AS1 levels were associated with advanced disease stages (stage III + IV), larger tumor size, and lymph node metastasis (Figures 1E–G). In LUAD cell lines (H1650, H1755, PC9, and H358), OIP5-AS1 expression was significantly lower than in normal human lung epithelial cells (BEAS-2B), as confirmed by qRT-PCR (Figure 1H). Among the LUAD cell lines, H358 showed the highest OIP5-AS1 expression, while H1650 had the lowest; therefore, and these 2 cell lines were selected for subsequent experiments.

### OIP5-AS1 overexpression suppressed LUAD cell proliferation and metastasis and induced G1 phase cell cycle arrest

**FIGURE 2:**
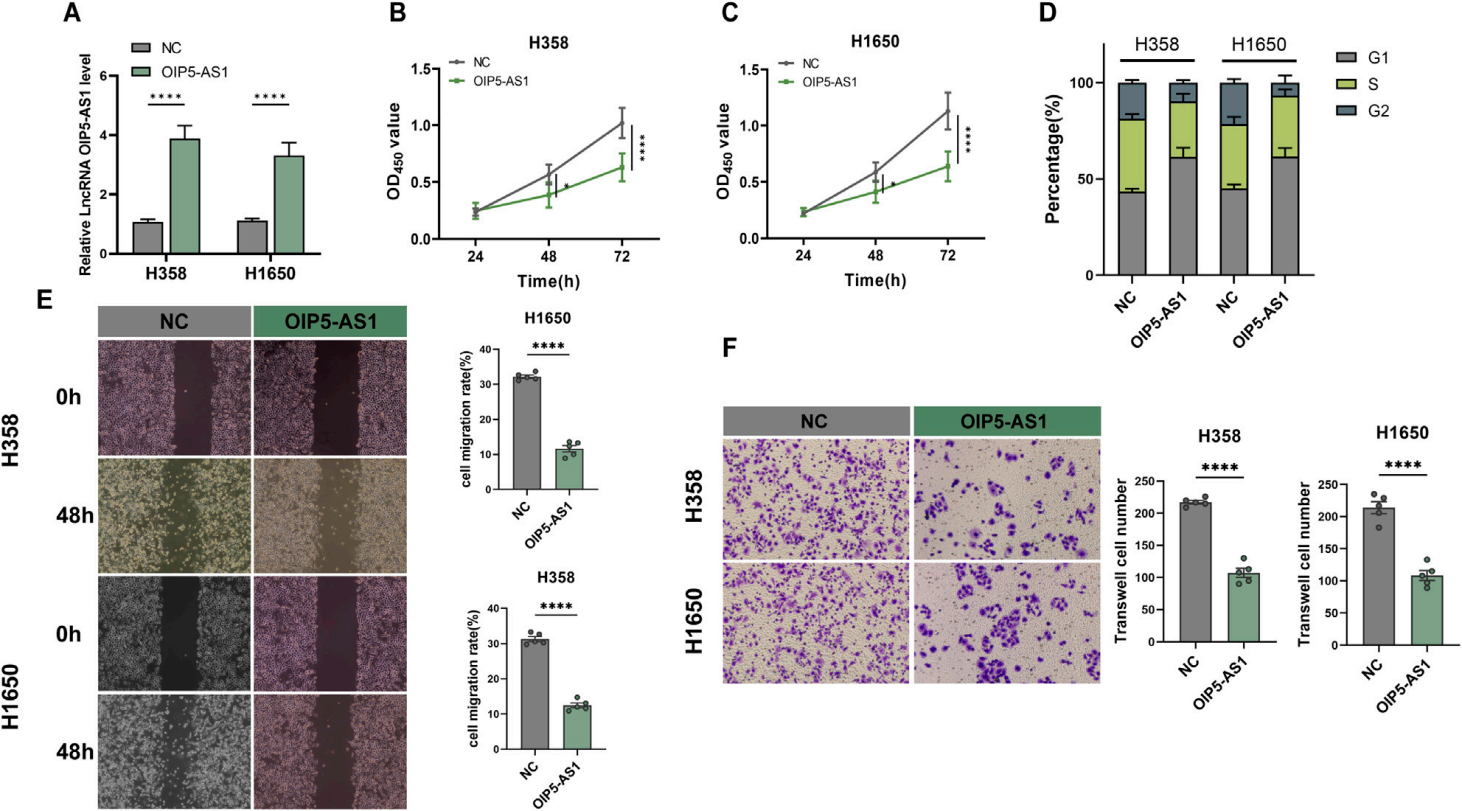
Functional evaluation of OIP5-AS1 in LUAD. **(A)** Relative expression of OIP5-AS1 in transfected cells was determined by qRT-PCR; **(B, C)** CCK8 was used to measure the proliferation of OIP5-AS1-transfected and control (NC) cells in H358 **(B)** and H1650 **(C)** cell lines; **(D)** Cell cycle distribution was analyzed by flow cytometry; **(E)** Cell migration evaluated using the wound healing assay (×100 magnification); **(F)** Cell invasion was evaluated using the Transwell assay (×200 magnification). (*p < 0.05, **p < 0.01, ***p < 0.001, ****p < 0.0001, NS: not significant).

To investigate the potential role of lncRNA OIP5-AS1 in LUAD cell function, H358 and H1650 cells were transfected to overexpress OIP5-AS1, with NC-transfected cells serving as controls. Transfection efficiency was confirmed by qRT-PCR (Figure 2A). The CCK-8 assay revealed that OIP5-AS1 overexpression reduced the viability of H358 and H1650 cells (Figures 2B, C). Flow cytometry further demonstrated that OIP5-AS1 overexpression induced G1 phase cell cycle arrest (Figure 2D; Supplementary Figure S1). Moreover, OIP5-AS1 overexpression significantly inhibited the migration and invasion abilities of H358 and H1650 cells (Figures 2E, F).

### OIP5-AS1 targeted miR-429

**FIGURE 3:**
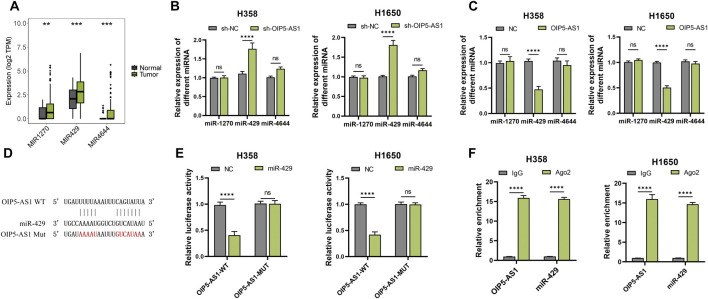
MiR-429 is a target of OIP5-AS1. **(A)** Expression levels of predicted target miRNAs in LUAD tumor and non-tumor tissues based on the TCGA dataset; **(B)** qRT-PCR analysis of target miRNA expression in OIP5-AS1 overexpression **(B)** or knockdown **(C)**; **(D)** Schematic representation of the predicted binding site between OIP5-AS1 and miR-429; **(E)** Luciferase reporter assay demonstrating the interaction between OIP5-AS1 and miR-429 in LUAD cells; **(F)** RIP assay with the anti-AGO2 antibody demonstrated the enrichment of OIP5-AS1 and miR-429. (*p < 0.05, **p < 0.01, ***p < 0.001, ****p < 0.0001, NS: not significant.).

LncRNAs are known to regulate cellular processes through diverse mechanisms, often acting as molecular sponges for miRNAs. It was hypothesized that OIP5-AS1 might function as a ceRNA by interacting with specific miRNAs in LUAD. Potential interactions between OIP5-AS1 and miRNAs were predicted using Starbase. TCGA analysis revealed that miR-1270, miR-429, and miR-4644 were significantly upregulated in LUAD tissues compared to normal tissues (Figure 3A). Among these, miR-429 exhibited the most pronounced upregulation (Figures 3B, C). Dual-luciferase reporter assays showed that co-transfection of miR-429 mimics with OIP5-AS1 WT reporter vectors significantly reduced luciferase activity, confirming a direct interaction between miR-429 and OIP5-AS1 (Figures 3D, E). This interaction was further validated by RIP assays, which demonstrated that both OIP5-AS1 and miR-429 were significantly enriched in the Ago2 pull-down group compared to the IgG control in H358 and H1650 cells (Figure 3F).

### miR-429 could target DOCK4 in LUAD

**FIGURE 4:**
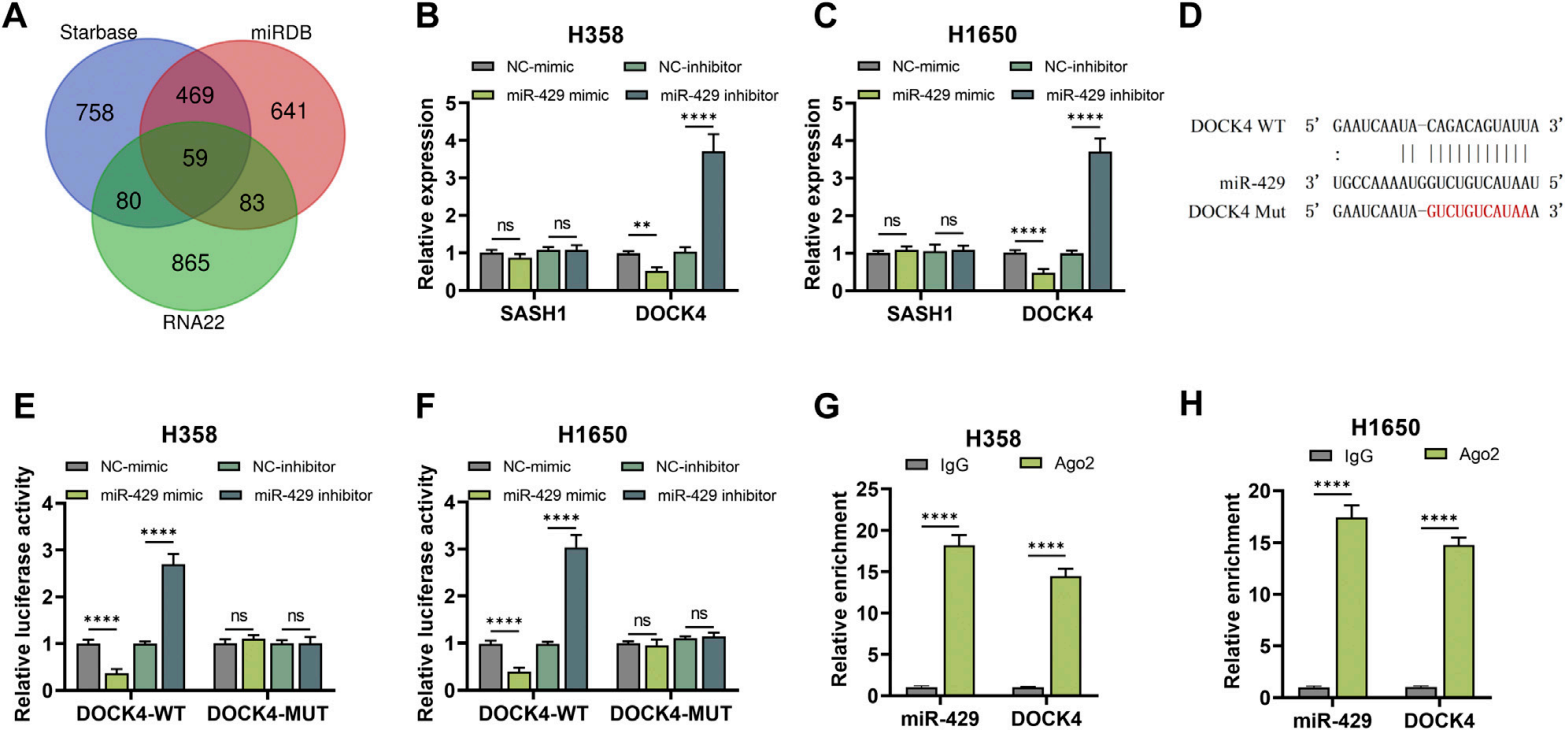
miR-429 directly binds to DOCK4. **(A)** Prediction tools (Starbase, miRDB, and RNA22) identified potential downstream targets of miR-429; **(B, C)** qRT-PCR analysis of SASH1 and DOCK4 mRNA expression in H358 **(B)** and H1650 **(C)** cells following miR-429 overexpression or knockdown; **(D)** Schematic representation of the predicted binding site between miR-429 and DOCK4; **(E, F)** Dual luciferase reporter assay confirmed the interaction between miR-429 and DOCK4 in H358 **(E)** and H1650 **(F)** cells; **(G, H)** RIP assay validated the interaction between miR-429 and DOCK4. (*p < 0.05, **p < 0.01, ***p < 0.001, ****p < 0.0001, NS: not significant.).

Potential targets of miR-429 were identified through the Starbase, miRDB, and RNA22 databases (Figure 4A). Among the predicted targets, SASH1 and DOCK4, which have been implicated in lung cancer prognosis, are recognized as oncogenes in LUAD (Liu A. et al., 2024; Burgess et al., 2020; Altaf et al., 2023). qRT-PCR analysis demonstrated that miR-429 overexpression significantly reduced, while its knockdown increased, the mRNA levels of DOCK4 (Figures 4B, C). Furthermore, luciferase reporter assays showed that the miR-429 mimic significantly decreased luciferase activity in DOCK4-WT constructs, whereas the miR-429 inhibitor had the opposite effect (Figures 4D–F). RIP assays confirmed the direct interaction, revealing that both miR-429 and DOCK4 were enriched in the Ago2 pull-down group compared to the IgG control (Figures 4G, H).These findings suggest that miR-429 directly interacts with DOCK4, regulating its expression.

### The biological role of OIP5-AS1 in LUAD cells was mediated through the miR-429/DOCK4 regulatory axis

**FIGURE 5:**
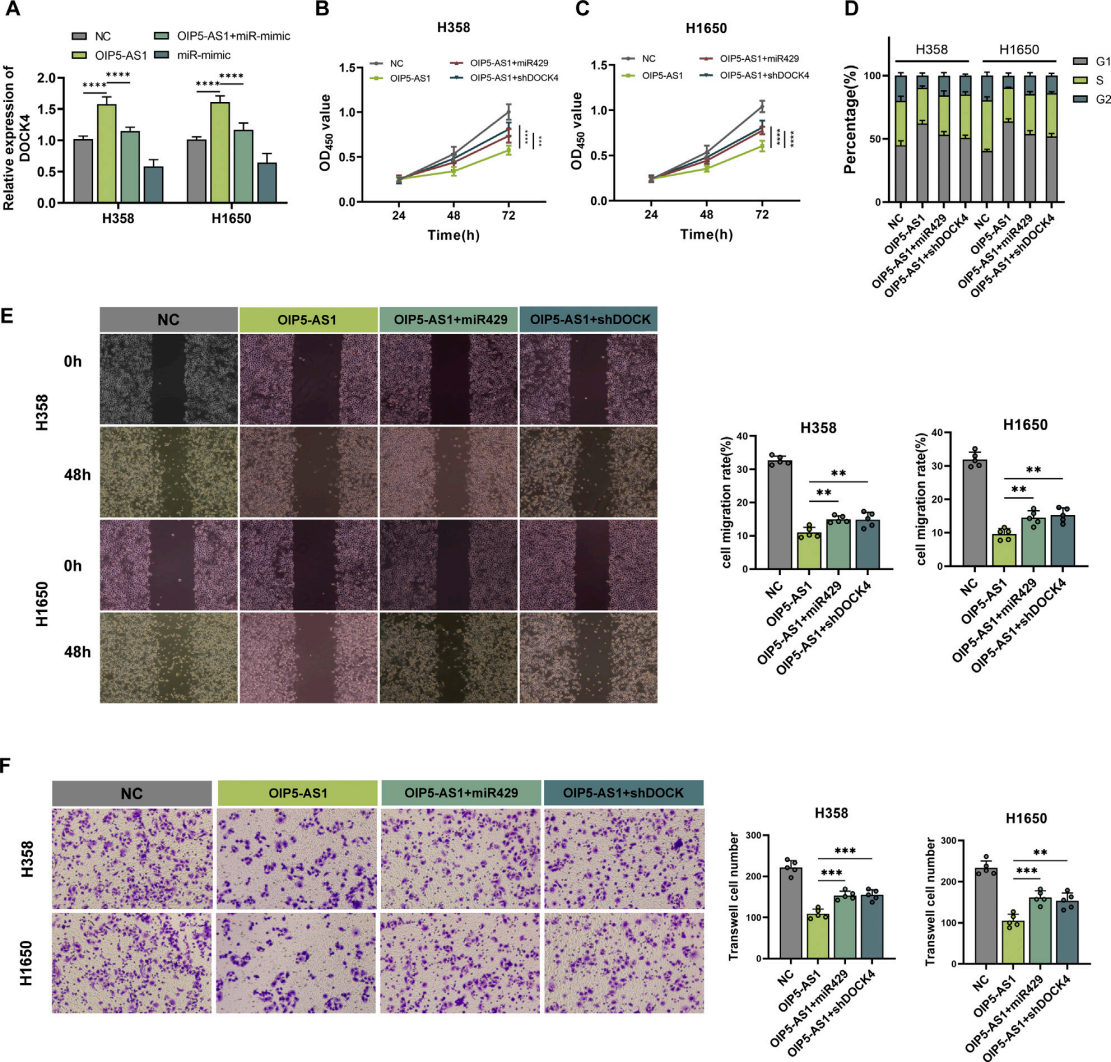
OIP5-AS1 inhibits cell growth and metastasis in LUAD via the miR-429/DOCK4 regulatory axis. **(A)** Relative expression levels of DOCK4 in transfected cells were measured by qRT-PCR; **(B, C)** Cell viability was assessed using the CCK-8 assay in H358 **(B)** and H1650 **(C)** cells; **(D)** Cell cycle distribution was analyzed by flow cytometry; **(E)** Cell migration was evaluated using the wound healing assay (×100 magnification); **(F)** Cell invasion was assessed using a Transwell assay (×200 magnification). (*p < 0.05, **p < 0.01, ***p < 0.001, ****p < 0.0001, NS: not significant.).

Analysis of the UALCAN and kmplotter tools revealed that DOCK4 expression was downregulated in LUAD tissues, and lower expression was associated with poor prognosis (Supplementary Figures S2-S3). To investigate whether OIP5-AS1 regulates LUAD progression via the miR-429/DOCK4 regulatory axis, miR-429 mimics or shDOCK4 plasmids were transfected into OIP5-AS1-overexpressing LUAD cells. OIP5-AS1 overexpression increased DOCK4 expression, an effect that was reversed by miR-429 mimics (Figure 5A). Furthermore, miR-429 overexpression and DOCK4 knockdown partially reversed the inhibitory effects of OIP5-AS1 on H358 and H1650 cell proliferation (Figures 5B, C). Flow cytometry showed that miR-429 overexpression and DOCK4 knockdown also partially reversed OIP5-AS1-induced G1 phase cell cycle arrest (Figure 5D; Supplementary Figure S4), and restored the migratory and invasive abilities of LUAD cells (Figures 5E, F). These results suggest that OIP5-AS1 functions as a ceRNA for miR-429 and may influence LUAD progression by modulating DOCK4 expression.

### OIP5-AS1/miR-429/DOCK4 regulatory axis regulated macrophage polarization

**FIGURE 6:**
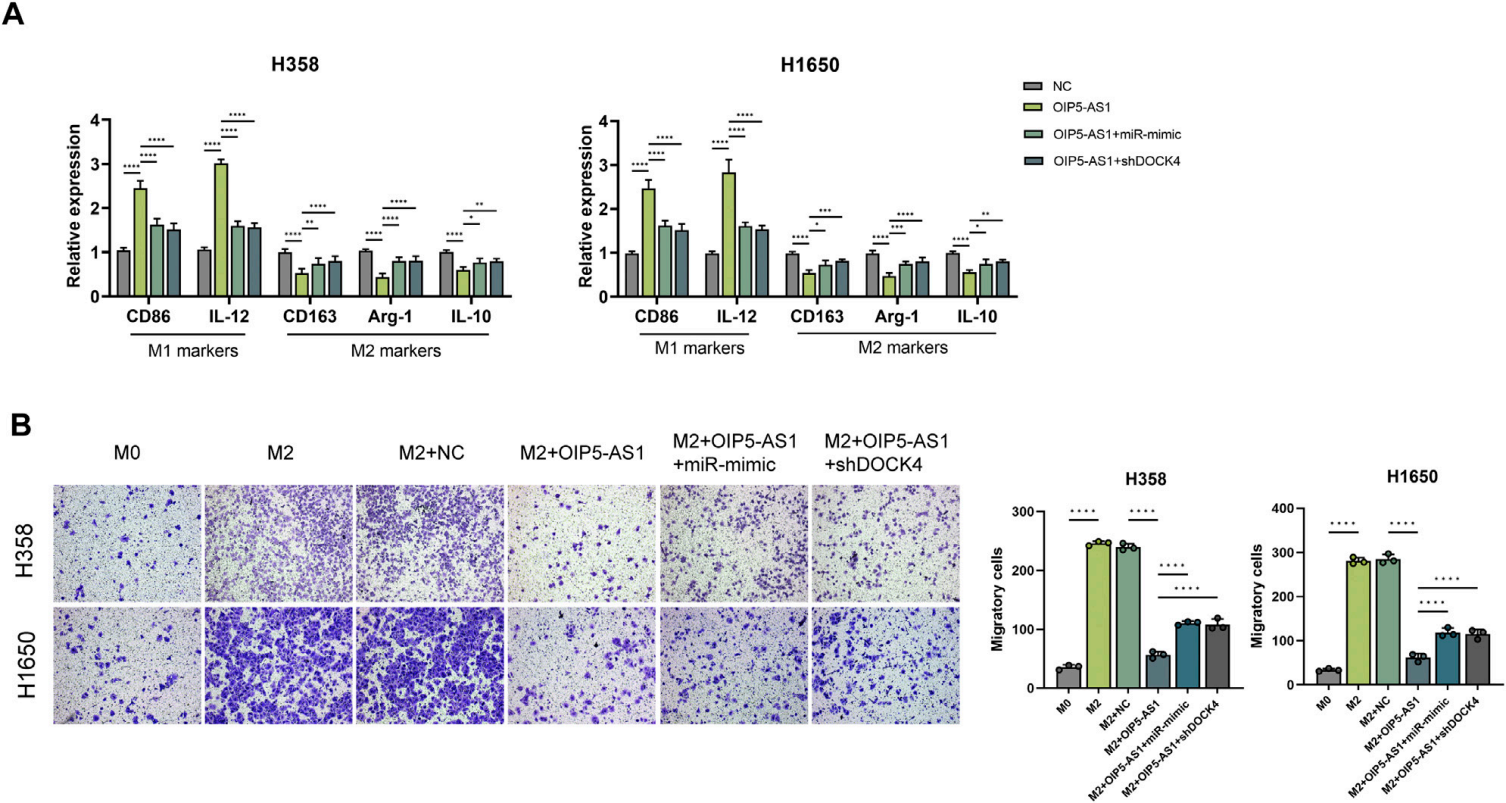
OIP5-AS1 suppresses M2 macrophage polarization by targeting the miR-429/DOCK4 regulatory axis. **(A)** Expression levels of macrophage polarization markers in co-cultures with tumor cells.; **(B)** Macrophage migration was assessed using chemotaxis assays under different conditions (*p < 0.05, **p < 0.01, ***p < 0.001, ****p < 0.0001, NS: not significant).

DOCK4 has been previously implicated in M2 tumor-associated macrophage (TAM) polarization and angiogenesis in lung adenocarcinoma, contributing to immune-related tumor functions and poor patient prognosis (Liu A. et al., 2024). To investigate the role of the OIP5-AS1/miR-429/DOCK4 regulatory axis in macrophage polarization, THP-1 cells were first differentiated into macrophages and then treated with conditioned medium derived from LUAD cells transfected with OIP5-AS1, miR-429, or DOCK4 constructs. qRT-PCR analysis revealed that OIP5-AS1 overexpression significantly downregulated M2 macrophage markers, including CD163, Arg-1, and IL-10, while upregulating M1 markers such as CD86 and IL-12. However, this effect was reversed by miR-429 mimic or DOCK4 knockdown (Figure 6A).

Chemotaxis assays showed that CM from OIP5-AS1-overexpressing LUAD cells significantly inhibited macrophages migration, whereas this effect was alleviated by miR-429 overexpression or DOCK4 knockdown in LUAD cells (Figure 6B).

## Discussion

The dysregulation of lncRNAs has been implicated in a wide range of biological and pathological processes, including cancer initiation and progression. Depending on the context, lncRNAs can act as oncogenes or tumor suppressors by modulating key regulatory networks (Zheng et al., 2022; Xie et al., 2024). This study identifies OIP5-AS1 as a tumor suppressor in LUAD that regulates the miR-429/DOCK4 regulatory axis, thereby inhibiting tumor cell proliferation, migration, and altering macrophage polarization within the TME.

Although OIP5-AS1 has been reported to function as an oncogene in several cancers, including colorectal (Wang et al., 2022b), gastric (Xie et al., 2024), and bladder cancers (Wang et al., 2019), it is downregulated in LUAD, as well as in endometrial (Liu et al., 2021) and ovarian cancers (Wang et al., 2022c). In the present study, OIP5-AS1 was downregulated in LUAD, which associated with advanced TNM stage, lymph node metastasis, and poor overall survival. These findings suggest a context-dependent role of OIP5-AS1, likely shaped by the TME, where it may exert tumor-suppressive effects by modulating both tumor cell behavior and immune cell function. Notably, tumor-associated macrophages (TAMs), particularly the M2 subtype, are key contributors to an immunosuppressive TME that facilitates tumor growth and metastasis (Boutilier and Elsawa, 2021; Zhang and Sioud, 2023; Li et al., 2020; Sarode et al., 2020). Our findings indicate that OIP5-AS1 plays a role in counteracting this effect by modulating macrophage polarization.

Mechanistically, OIP5-AS1 acts as a ceRNA for miR-429, an oncogenic miRNA implicated in several cancers, including breast (Li et al., 2023) and colorectal cancer (Li et al., 2013). In this study, the interaction between OIP5-AS1 and miR-429 was confirmed by bioinformatics prediction, dual-luciferase reporter assays, and RIP assays. Functional experiments showed that miR-429 overexpression reversed the anti-proliferative and anti-migratory effects of OIP5-AS1, as well as G1 phase cell cycle arrest, supporting the notion that OIP5-AS1 suppresses LUAD progression through sponging miR-429 and inhibiting its oncogenic activity.

Through database predictions and experimental validation, DOCK4 was identified as a downstream target of miR-429. Analyses of UALCAN and kmplotter datasets revealed that lower DOCK4 expression in LUAD samples is associated with unfavorable patient outcomes, consistent with previous reports (Yu et al., 2020)。Functionally, DOCK4 has been reported to regulate tumor cell migration, invasion, and metastasis in LUAD, primarily through Rac1 activation under TGF-β/Smad signaling (Yu et al., 2015). Given that Rac1 also plays a key role in the migration of immune cells such as macrophages (Liang J. et al., 2021), it is plausible that DOCK4 may influence macrophage trafficking within the tumor microenvironment.

Our experimental results support this notion. Overexpression of OIP5-AS1 in co-culture systems resulted in a marked downregulation of M2 macrophage markers (CD163, Arg-1, and IL-10), while this effect was reversed by miR-429 overexpression or DOCK4 knockdown. These findings suggest that DOCK4 is involved in macrophage polarization and may mediate the inhibitory effects of OIP5-AS1 on M2-type TAMs. In addition, chemotaxis assays showed that conditioned medium from OIP5-AS1-overexpressing significantly suppressed macrophage migration, whereas this inhibitory effect was attenuated by either miR-429 mimic or DOCK4 silencing. These results suggest that the OIP5-AS1/miR-429/DOCK4 regulatory axis not only affects macrophage polarization but also modulates their migratory behavior, which may in turn influence immune cell infiltration and spatial distribution within the tumor microenvironment.

Importantly, previous studies have reported that DOCK4 is associated with M2-type TAMs in LUAD, further supporting its role as a potential immune-related prognostic marker (Liu A. et al., 2024). Together with our findings, this highlights the dual role of DOCK4 in both tumor-intrinsic and immune-regulatory processes.

Interestingly, the functional role of DOCK4 appears to be context-dependent. Similar to other DOCK family members such as DOCK2—which exhibits either tumor-suppressive or tumor-promoting roles depending on the specific tumor context and immune microenvironment—DOCK4 is involved in diverse signaling pathways in LUAD, including MYC signaling and DNA repair (Wu et al., 2017; Hu et al., 2019; Zeng et al., 2021). This complexity suggests that DOCK4 may act as an integrative node influenced by external cues and tumor microenvironmental factors, contributing to the dynamic regulation of both tumor progression and immune modulation.

Overall, these findings support a model in which OIP5-AS1 regulates macrophage polarization and migration through the miR-429/DOCK4 regulatory axis. This regulatory pathway appears to exert dual effects by modulating both tumor cell behavior and the tumor immune microenvironment, offering novel insights into the mechanisms underlying LUAD progression and potential therapeutic strategies aimed at enhancing antitumor immunity.

Clinically, assessing OIP5-AS1 expression in a larger cohort of LUAD patients could provide valuable prognostic information, aiding in patient risk stratification and informing treatment decisions. Given the growing interest in targeting noncoding RNAs in cancer therapy, further investigations are warranted to determine whether modulating OIP5-AS1 expression could offer therapeutic benefits in LUAD.

Mechanistically, our study identifies DOCK4 as a potential upstream regulator of macrophage polarization; however, the precise mechanisms through which it exerts this effect remain to be fully elucidated. As a guanine nucleotide exchange factor (GEF), DOCK4 primarily regulates intracellular signaling rather than acting as a direct secretory mediator. Therefore, further investigations are required to determine whether DOCK4 influences macrophage polarization through the modulation of tumor-derived cytokines and chemokines. Future studies will explore the specific soluble mediators involved by profiling the secretome of DOCK4-overexpressing LUAD cells and assessing the functional impact of targeted cytokine inhibition. Additionally, flow cytometry analysis of macrophage surface markers will be performed to provide a more comprehensive assessment of M1/M2 polarization in response to DOCK4 modulation.

Moreover, *in vivo* studies will be essential to determine whether OIP5-AS1-mediated macrophage polarization can effectively suppress LUAD progression and to evaluate the therapeutic efficacy of modulating this pathway in a physiological context.

From a therapeutic perspective, recent studies have proposed several feasible approaches for targeting noncoding RNA pathways, including siRNA- or antisense oligonucleotide (ASO)-based strategies, as well as natural compounds capable of modulating specific miRNA networks (Chen et al., 2021; Huang et al., 2017). These advances support the potential clinical applicability of targeting the OIP5-AS1/miR-429/DOCK4 regulatory axis. However, further investigations are needed to validate these findings *in vivo* and to clarify the therapeutic efficacy and safety of manipulating OIP5-AS1 expression or its upstream regulators.

In summary, this study identifies OIP5-AS1 as a key regulator of LUAD progression through the miR-429/DOCK4 regulatory axis, affecting both tumor-intrinsic pathways and macrophage-mediated immune modulation. These findings provide a basis for future research into RNA-based therapeutic strategies targeting the tumor–immune microenvironment in LUAD.

## Data Availability

The original contributions presented in the study are included in the article/Supplementary Material, further inquiries can be directed to the corresponding authors.
