# Supplementary material for: LncRNA OIP5-AS1 suppresses lung adenocarcinoma progression and modulates macrophage polarization through the miR-429/DOCK4 regulatory axis
Source: Front Pharmacol. 2025 May 20;16:1569644. doi: 10.3389/fphar.2025.1569644 (PMC12171188; doi:10.3389/fphar.2025.1569644)
Supplement: Supplementary file 1 [file Table1.docx]

**LncRNA OIP5-AS1 suppresses lung adenocarcinoma progression and modulates macrophage polarization through the miR-429/DOCK4 regulatory axis**

Yihan Liu^1†^,Yuhua Wang^2†^, Long Cui^3†^, Runze Li^4^, Dan Xiao^4,5^*

^1^Graduate School, Heilongjiang University of Chinese Medicine, Harbin 150040, China

^2^School of Marxism, Qiqihar Medical University, Qiqihar 161006, China

^3^Department of Oncology, Qiqihar Hospital of Chinese Medicine, Qiqihar 161000, China.

^4^National and Local Joint Engineering Laboratory for Synthesis Transformation and Separation of Extreme Environmental Nutrients, Harbin Institute of Technology, Harbin 150001, China

^5^Zhengzhou Research Institute, Harbin Institute of Technology, Zhengzhou 450007, China

*Corresponding author to Dan Xiao: Associated professor, School of Medicine and Health, Harbin Institute of Technology, No. 92, Xidazhi Street, Nangang District, Harbin, Heilongjiang, 150001, China. E-Mail: xiaodan@hit.edu.cn

**†**These authors contributed equally to this work.

**Supplemental Table S1 The target sequences of short hairpin RNAs (shRNA)**

| **Target gene** | **TRC Clone ID** | **Target region** | **Target sequence** |
| --- | --- | --- | --- |
| OIP5-AS1 | TRCN0000162795 | 3’-UTR | CTTCCAAAGTGCTGGGATTAT |
| DOCK4 | TRCN0000039731 | CDS | GCTTCGAGTTTCGGCATTGTT |

**Supplementary Table S2 The primers of the detected genes**

| **gene** |  | **Primer（5’-3’）** |
| --- | --- | --- |
| OIP5-AS1 | Forward | TGCGAAGATGGCGGAGTAAG |
|  | Reverse | CAACAGGCGTCTTGGTGTTG |
| GAPDH | Forward | ACCACAGTCCATGCCATCAC |
|  | Reverse | TCCACCACCCTGTTGCTGTA |
| U6 | Forward | CGCTAGCACATATCGGCTA |
|  | Reverse | TTCTGCGACGAATTTGTCAT |
| miR-429 | Forward | CGCGCGTAATACTGTCTGGTAA |
|  | Reverse | AGTGCAGGGTCCGAGGTATT |
| miR-1270 | Forward | CTGGAGATATGGAAGAGCT |
|  | Reverse | CAGTGCGTGTCGTGGAGT |
| miR-4644 | Forward | GTCGTATCCAGTGCAGGGTCCGAGGT |
|  | Reverse | GCGTGGAGAGAGAAAAGAGA |
| DOCK4 | Forward | GACCCACACACAGACTGCTTCA |
|  | Reverse | GAGAGGGGGTGAAAGACTGC |
