# Supplementary material for: LncRNA OIP5-AS1 suppresses lung adenocarcinoma progression and modulates macrophage polarization through the miR-429/DOCK4 regulatory axis
Source: Front Pharmacol. 2025 May 20;16:1569644. doi: 10.3389/fphar.2025.1569644 (PMC12171188; doi:10.3389/fphar.2025.1569644)
Supplement: Supplementary file 2 [file DataSheet1.docx]

**LncRNA OIP5-AS1 suppresses lung adenocarcinoma progression and modulates macrophage polarization through the miR-429/DOCK4 regulatory axis**

Yihan Liu^1†^,Yuhua Wang^2†^, Long Cui^3†^, Runze Li^4^, Dan Xiao^4,5^*

^1^Graduate School, Heilongjiang University of Chinese Medicine, Harbin 150040, China

^2^School of Marxism, Qiqihar Medical University, Qiqihar 161006, China

^3^Department of Oncology, Qiqihar Hospital of Chinese Medicine, Qiqihar 161000, China.

^4^National and Local Joint Engineering Laboratory for Synthesis Transformation and Separation of Extreme Environmental Nutrients, Harbin Institute of Technology, Harbin 150001, China

^5^Zhengzhou Research Institute, Harbin Institute of Technology, Zhengzhou 450007, China

*Corresponding author to Dan Xiao: Associated professor, School of Medicine and Health, Harbin Institute of Technology, No. 92, Xidazhi Street, Nangang District, Harbin, Heilongjiang, 150001, China. E-Mail: xiaodan@hit.edu.cn

**†**These authors contributed equally to this work.

**Supplementary Figure**

**
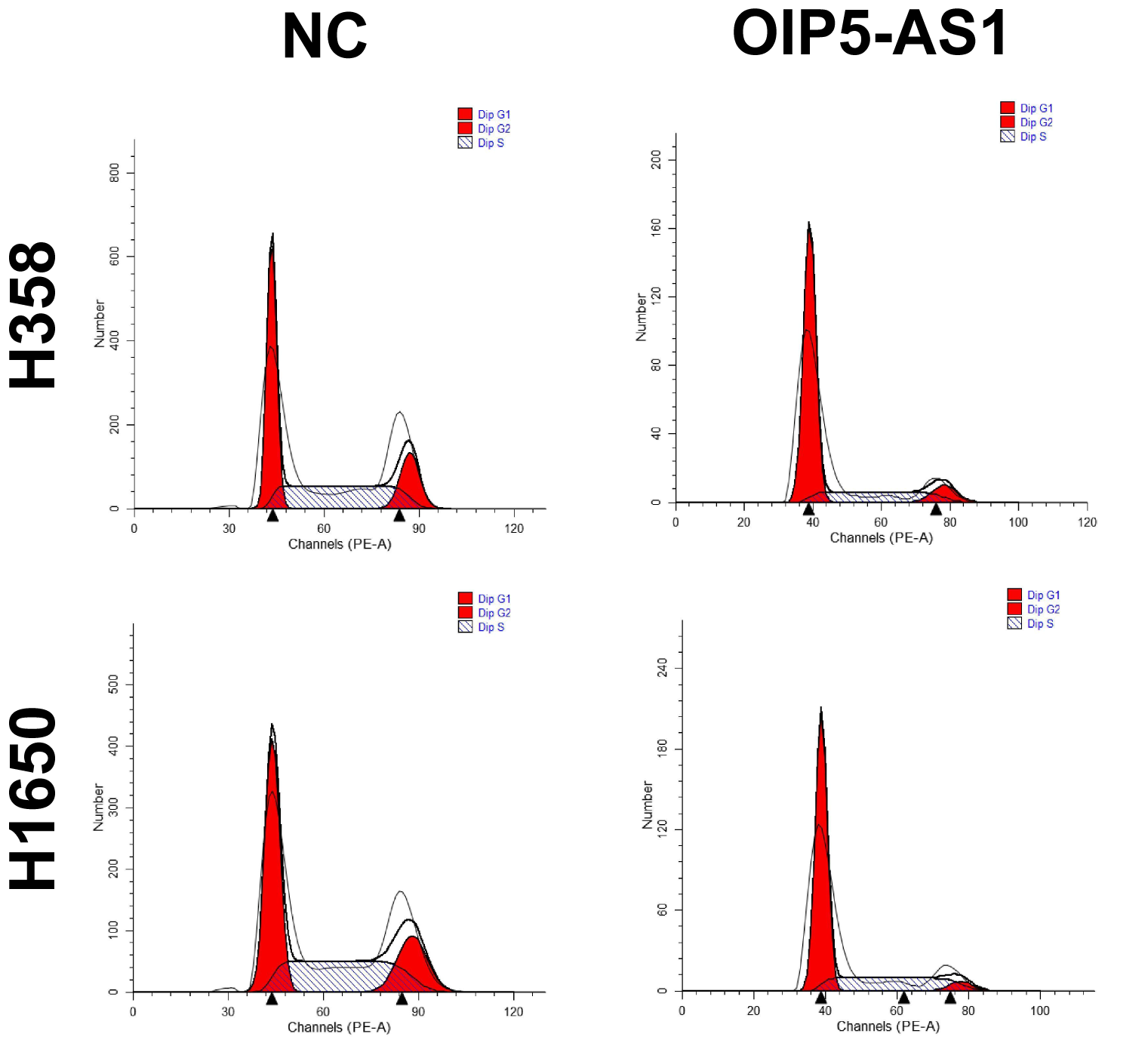
**

**Supplementary Figure S1 ) Cell cycle distribution was analyzed by flow cytometry.**


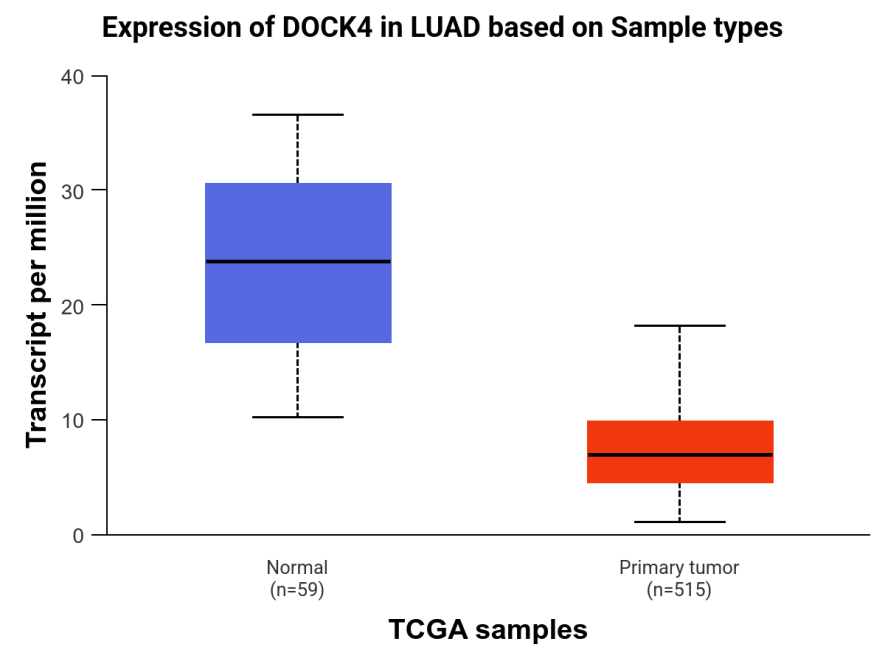


**Supplementary Figure S2 Expression of DOCK4 in LUAD tissues and normal tissues analyzed using the UALCAN tool based on TCGA-LUAD data.**


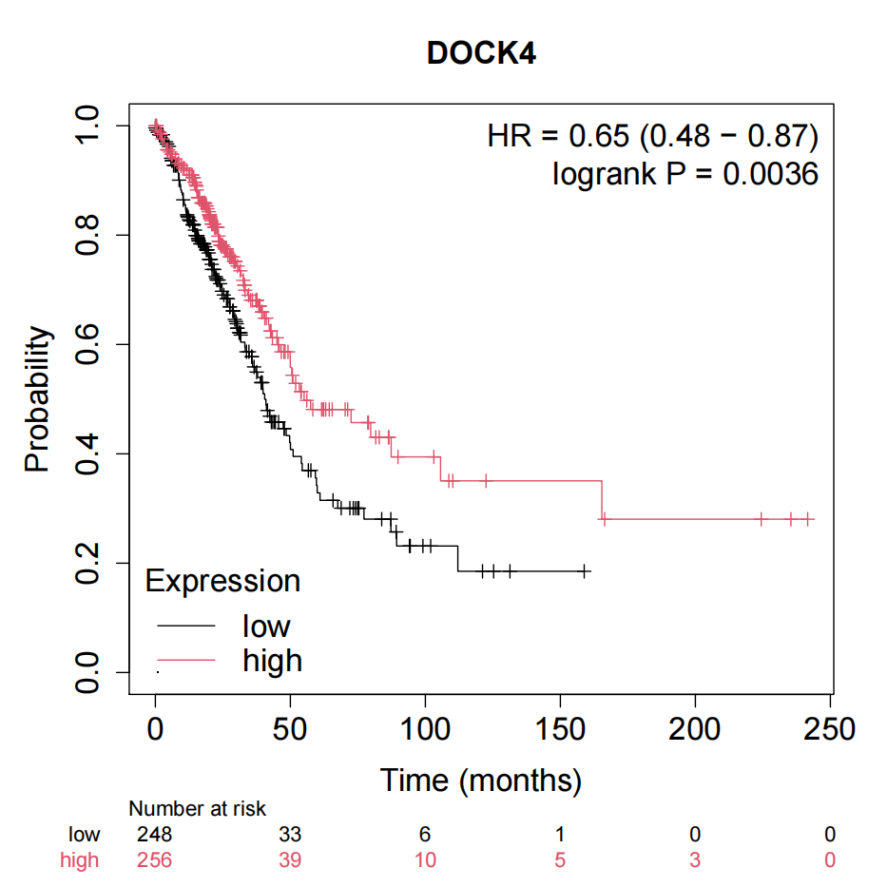


**Supplementary Figure S3 Prognostic significance of DOCK4 expression in LUAD analyzed using the KM plotter tool.**

**
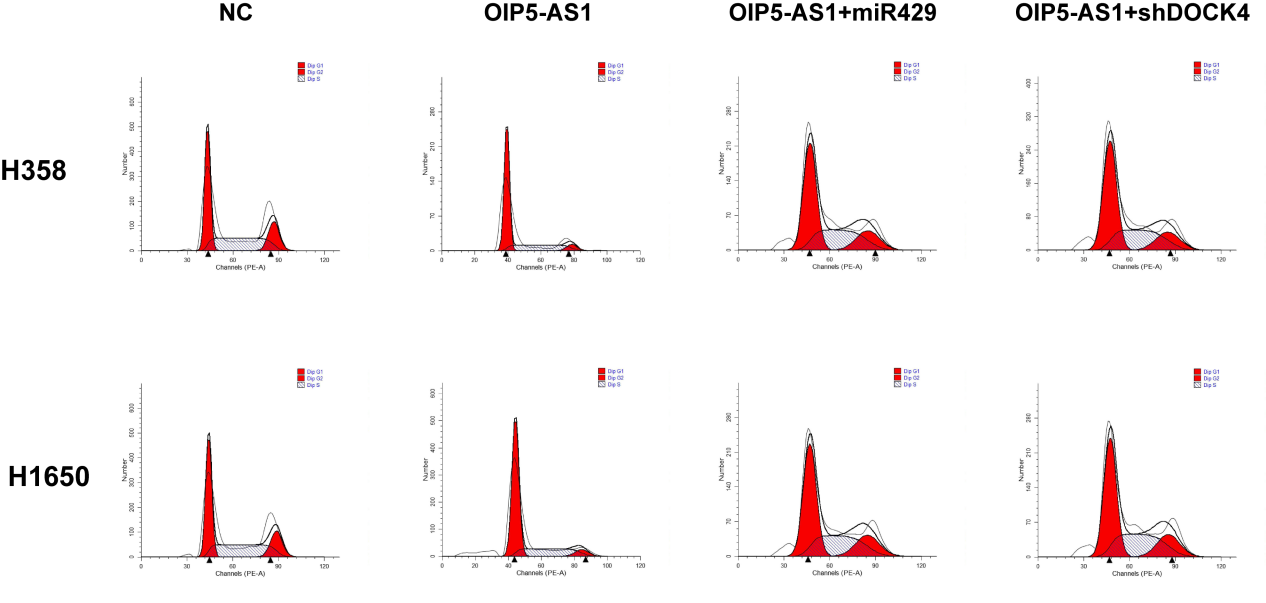
**

**Supplementary Figure S4 Cell cycle distribution was analyzed by flow cytometry**
